# Molecular Characterization and Pathogenicity of Watermelon Isolates of *Begomovirus cucurbitachinaense*

**DOI:** 10.3390/ijms26094289

**Published:** 2025-05-01

**Authors:** Liming Liu, Yanhui Wang, Yanfei Geng, Bo Yu, Leiyan Yan, Fangmin Hao, Huijie Wu, Pingyong Wang, Qinsheng Gu, Baoshan Kang

**Affiliations:** 1 Henan Key Laboratory of Fruit and Cucurbit Biology, Zhengzhou Fruit Research Institute, Chinese Academy of Agricultural Sciences, Zhengzhou 450009, China; liuliming@caas.cn (L.L.); wangyanhui061@163.com (Y.W.); gengyanfei2017@163.com (Y.G.); wuhuijie@caas.cn (H.W.); wangpingyong@caas.cn (P.W.); guqinsheng@caas.cn (Q.G.); 2 Zhengzhou Agricultural Science and Technology Research Institute, Zhengzhou 450005, China; 3 Zhongyuan Research Center, Chinese Academy of Agricultural Sciences, Xinxiang 453500, China; 4 College of Horticulture, Henan Agricultural University, Zhengzhou 450002, China; 18339998024@163.com; 5 Ningbo Key Laboratory of Characteristic Horticultural Crops in Quality Adjustment and Resistance Breeding, Ningbo Academy of Agricultural Sciences, Ningbo 315040, China; yanleiyan@zju.edu.cn (L.Y.); haofangmin@163.com (F.H.)

**Keywords:** watermelon, squash leaf curl China virus, whitefly transmission, infectious clone

## Abstract

Squash leaf curl China virus (SLCCNV) belongs to the species *Begomovirus cucurbitachinaense* in the genus *Begomovirus* and can infect some Cucurbitaceae crops except for watermelon (*Citrullus lanatus*). In this study, watermelon plants showing symptoms typical to begomovirus infection in field were observed in Zhejiang Province of China, and SLCCNV presence was identified through PCR and next-generation sequencing (NGS). The pairwise sequence identity of the DNA-A genome shows that SLCCNV watermelon isolate belongs to the SLCCNV/CN strain and shares 96% nucleotide identity with the previously sequenced SLCCNV/CN. An infectious clone of SLCCNV watermelon isolate was constructed using the tandem repeat fragment method. Through agrobacterium-mediated inoculation, the clone could induce systemic infection with typical symptoms in watermelon, melon (*Cucumis melo*), squash (*Cucurbita pepo*), pumpkin (*Cucurbita maxima*), wax gourd (*Benicasa hispida*), cucumber (*Cucumis sativus*), and *N. benthamiana*. It was further demonstrated that the progeny virions derived from the cloned watermelon isolate could be transmitted by whitefly rather than the sap. To the best of our knowledge, this is the first report of a natural infection of SLCCNV on watermelon in China, and the first complete report on the molecular characteristics and pathogenicity of watermelon-infecting SLCCNV in the world.

## 1. Introduction

Geminiviruses cause devastating diseases in many economically important crops and economic losses worldwide. The family *Geminiviridae*, with a total of over 500 species, is classified into 14 genera including *Becurtovirus*, *Begomovirus*, *Capulavirus*, *Citlodavirus Curtovirus*, *Eragrovirus*, *Grablovirus*, *Maldovirus Mastrevirus*, *Mulcrilevirus*, *Opunvirus*, *Topilevirus*, *Topocuvirus*, and *Turncurtovirus*, based on insect vectors, genome organizations, and host ranges [1]. *Begomovirus* is a genus with the most species (445 species and 8 unclassified species) [2]. They exclusively infect dicots, and the genome can be either bipartite, which contains two circular ssDNA molecules, DNA-A and DNA-B [3], or monopartite, which contains a single circular ssDNA molecule, DNA-A [4]. The DNA-A encodes all the proteins required for viral replication and encapsidation, including AV1 (capsid protein, CP), AV2, AC1 (replication-associated protein, Rep), AC2 (transcriptional activator protein, TrAP), AC3 (replication enhancer protein, REn), and AC4. Few begomoviruses encode AC5 [5]. The DNA-B encodes BV1 (nuclear shuttling protein, NSP) and BC1 (movement protein, MP). DNA-A can replicate autonomously and is responsible for generating complementary-sense (CS) and virion-sense (VS) strands through rolling-loop replication and recombination-dependent replication and producing virion [6,7]. DNA-B cannot replicate autonomously but is required for systemic infection and symptom development [8,9]. Monopartite begomoviruses are frequently accompanied by betasatellites, alphasatellites, or deltasatellites, and betasatellites have been confirmed to be associated with symptom induction of the disease [10,11,12]. Begomoviruses are transmitted by whiteflies (*Bemisia tabaci*) in a persistent circulative manner, and the specificity of whitefly vector is associated with specific amino acid sequences of the viral CP [13]. In addition, it has been proven that for tomato yellow leaf curl virus (TYLCV), replication occurs not only in the host plant but also in the salivary glands of whiteflies [14].

Squash leaf curl China virus (SLCCNV) belongs to the species *Begomovirus cucurbitachinaense* in the genus *Begomovirus*. It has a typical genome structure of begomoviruses, and the DNA-A encodes AC5, which acts as the RNA-silencing inhibitor that can suppress posttranscriptional gene silencing (PTGS) induced by ssRNA instead of dsRNA, and reverse GFP silencing and suppress GFP systemic silencing by interfering with the systemic spread of GFP silencing signals [15]. For vector transmission of SLCCNV, the minimum acquisition period (AAP) and inoculation feeding period (IFP) required for whiteflies are both determined to be 10 min, and sex difference in whiteflies affects the efficiency of virus transmission [16]. SLCCNV has a narrow host range, mainly infecting cucurbits crops, including pumpkin (*Cucurbita maxima*), causing yellow vein mosaic or yellow leaf curl disease [17,18]; wax gourd (*Benicasa hispida*), with severe disease incidence, causing crop losses of up to 100% [19]; and melon (*Cucumis melo*), causing yellowing, leaf curling, and dwarfing diseases with the incidence of disease being up to 40% [20]. In addition, SLCCNV has been reported to infect tomato (*Solanum lycopersicum*) [21], wild eggplant (*Solanum torvum*) [22], and *Bolbostemma paniculatum* [23] in China. Although reports indicate that watermelon samples collected in Indonesia contained SLCCNV, the study did not perform further whole-genome characterization of the watermelon isolates [24]. So far, the relationship between SLCCNV isolates and hosts has not been elucidated by inoculating SLCCNV isolates onto hosts other than the natural host.

In the autumn of 2023, during a disease investigation in Ningbo, Zhejiang, China, it was found that watermelons (*Citrullus lanatus*) grown in greenhouses exhibited symptoms such as small leaves, mosaic, yellowing, and leaf curling, which is very similar to the typical symptoms of begomoviruses. Previous reports have shown that watermelon can be infected by many begomoviruses, such as cucurbit leaf crumple virus (CuLCrV) [25], watermelon chlorotic stunt virus (WmCSV) [26], melon chlorotic mosaic virus (MeCMV) [27], squash leaf curl virus (SLCuV) [28], chili leaf curl virus (ChiLCV) [29], and tomato leaf curl New Delhi virus (ToLCNDV) [3]. In order to determine whether watermelon yellow leaf curl diseases are caused by begomoviruses, in this study, we first identified the viruses present in diseased watermelon through PCR and next-generation sequencing (NGS). Subsequently, we confirmed that SLCCNV is the pathogen of the disease and clarified the relationship between watermelon diseases and SLCCNV by constructing and inoculating an infectious clone. In addition, the capabilities of mechanical and vector transmissions, and the infectivity of SLCCNV watermelon isolate on other crops were analyzed to fully understand its biological characteristics.

## 2. Results

### 2.1. Discovery of Squash Leaf Curl China Virus in Watermelon Diseased Leaves Through Next-Generation Sequencing (NGS) and PCR Identification

**Figure 1:**
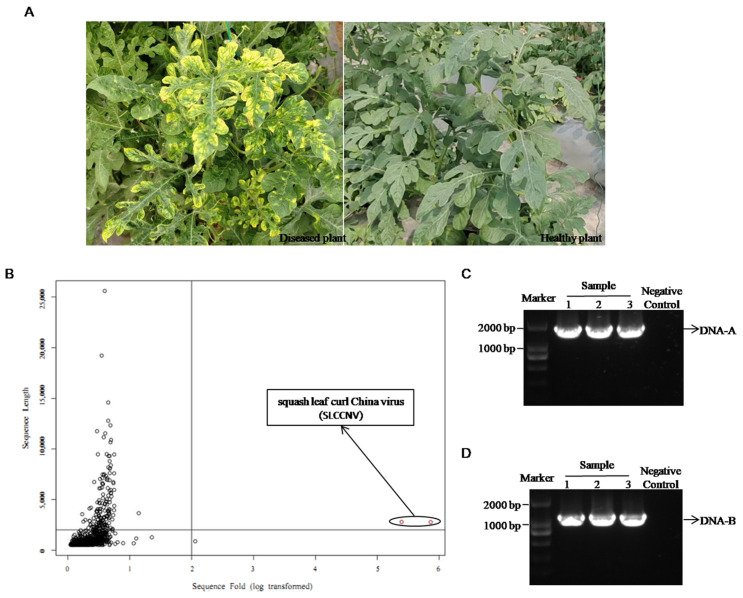
Symptoms shown in watermelon (**A**) and discovery of squash leaf curl China virus (SLCCNV) in watermelon diseased leaves through next-generation sequencing (NGS) (**B**) and PCR identification (**C**,**D**). (**B**) The result of scaffold lengths versus (vs.) coverage of all assembled fragments. The first quadrant contains all suspicious sequences with high content > 2 kb and only two fragments in the bottom right corner are high content fragments, both of which are SLCCNV. The “negative control” in (**C**,**D**) refers to sample collected from healthy plants in plastic greenhouses.

Three leaves exhibiting mosaic, yellowing, and curling from different watermelon plants (Figure 1A) were mixed for NGS analysis. After removing the host species sequence, the sequences were reassembled and annotated. The result discovered a high content of known begomovirus with a bipartite genome, squash leaf curl China virus (SLCCNV) (Figure 1B), in the tested sample. Based on the sequencing results, specific primers A304F/A1848R and B1092F/B2180R were used to detect SLCCNV DNA-A and DNA-B in the above three leaves. The electrophoresis results showed that all three samples contained SLCCNV (Figure 1C,D), consistent with the NGS results. 

### 2.2. Complete Genome Sequences of SLCCNV Watermelon Isolates and Sequence Identity Analysis

**Figure 2:**
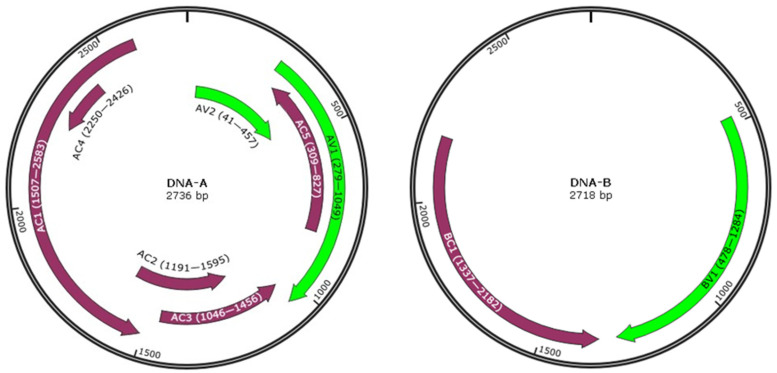
Genomic organizations of the SLCCNV watermelon (WM) isolates DNA-A and DNA-B. The virion-sense (VS) strand encodes AV1 (capsid protein—CP), AV2, and BV1 (nuclear shuttling protein—NSP); the complementary-sense (CS) strand encodes AC1 (replication-associated protein—Rep), AC2 (transcriptional activator protein—TrAP), AC3 (replication enhancer protein—REn), AC4, AC5, and BC1 (movement protein—MP); and the regions in the genome are displayed.

Four pairs of specific primers were used to amplify SLCCNV in three watermelon samples, and complete genome sequences of three SLCCNV watermelon isolates WM1, WM2 and WM3 were obtained. The DNA-A genome of isolates WM1~3 are all 2736 bp in length, with GenBank accession numbers PP886071, PP886072, and PP886073, respectively. The DNA-B genome of isolates WM1~3 are all 2718 bp in length, with GenBank accession numbers PP886074, PP886075, and PP886076, respectively. DNA-A encodes seven proteins containing AV1, AV2, AC1, AC2, AC3, AC4, and AC5, and DNA-B encodes two proteins containing BV1 and BC1. The detailed genome organization is shown in Figure 2. Sequence identity analysis was performed among isolates WM1~3. The nucleotide sequences of DNA-A and DNA-B among isolates WM1~3 shared 99.8–99.9% identities, and the pairwise sequence identity of DNA-A between isolates WM1~3 and other SLCCNV representative stains members were 96.0% for SLCCNV/CN (GenBank accession number: AF509743), 92.0% for SLCCNV (GenBank accession number: EU487031), 89.9% for SLCCNV/IN (GenBank accession number: AM286794), and 93.9-94.0% for SLCCNV/TH (GenBank accession number: EU543562), suggesting that isolates WM1~3 belong to the SLCCNV/CN strain of the specie *Begomovirus cucurbitachinaense*, with more than 94% pairwise sequence identity according to the strain demarcation criteria of the genus *Begomovirus* [30].

Furthermore, the analysis of both nucleotide and amino acid sequence identity was performed between SLCCNV WM1 and other isolates (Table 1). The results showed that the nucleotide sequence identities between WM1 and other SLCCNV isolates were 88.8–99.2% for DNA-A and 81.5–98.3% for DNA-B. At the amino acid sequence level, the identities between WM1 and other SLCCNV isolates were 80.4–99.6% for AV1, 55.7–99.2% for AV2, 72.6–98.8% for AC1, 66.4–97.7% for AC2, 81.6–98.5% for AC3, 79.3–96.5% for AC4, 68.0–99.4% for AC5, 79.3–99.6 for BC1, and 83.2–99.6 for BV1. Among them, the nucleotide sequence of DNA-A of isolate WM1 shared the highest identity (99.2%) with melon isolate HA3 from Hainan, China, and the nucleotide sequence of DNA-B of isolate WM1 shared the highest identity (98.3%) with pumpkin isolate GDHY from Guangdong, China.

### 2.3. Phylogenetic Analysis

**Figure 3:**
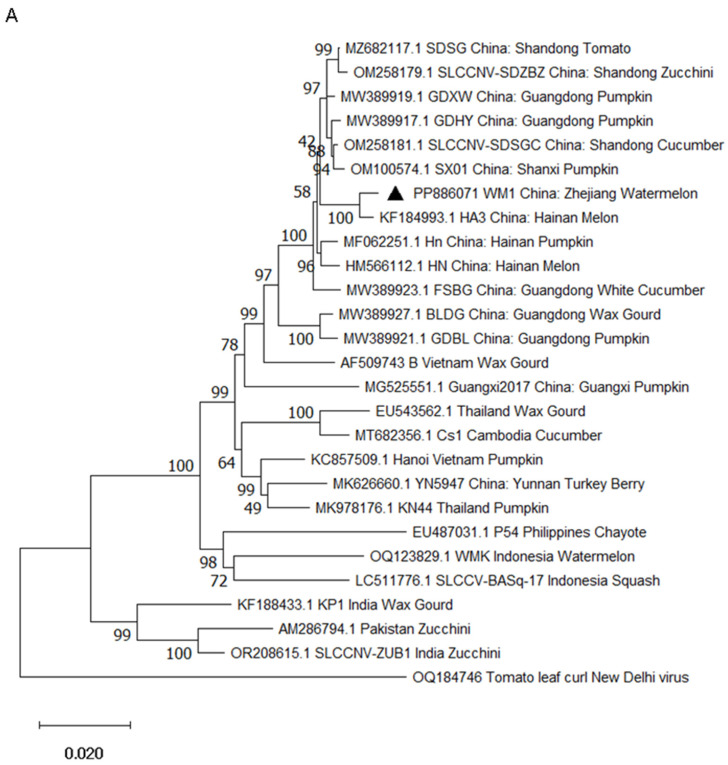

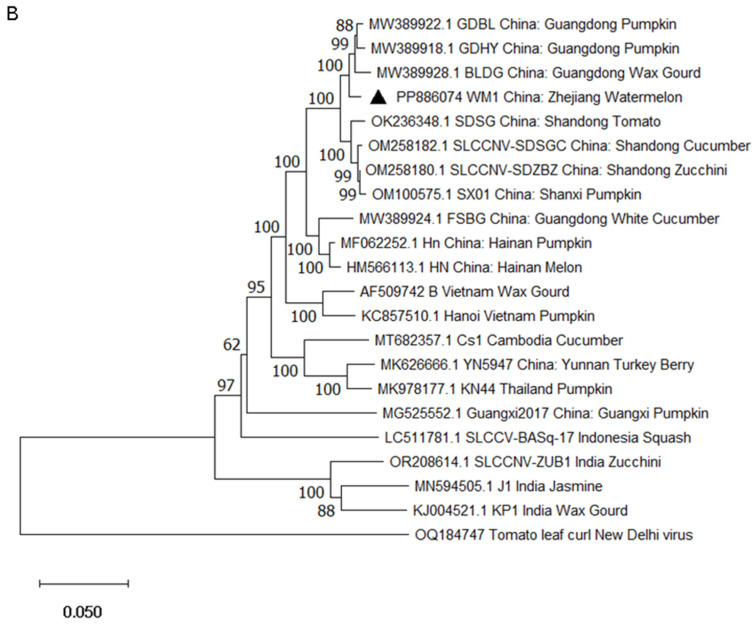
Phylogenetic trees based on alignment of the complete genome sequences of DNA-A (**A**) and DNA-B (**B**) of SLCCNV WM1 and other isolates by using the neighbor-joining method with 1000 bootstrap replicates with the MEGA X [31]. Tomato leaf curl New Delhi virus (ToLCNDV) is a member of the genus *Begomovirus* and was used as an outgroup. The SLCCNV WM1 isolate obtained in this study was labeled with “▲”.

Phylogenetic analyses based on the complete genome sequences of DNA-A of different SLCCNV isolates revealed WM1 isolate grouped with SDSG, SLCCNV-SDZBZ, GDXW, GDHY, SLCCNV-SDSGC, SX01, HA3, Hn, HN, FSBG, BLDG, and GDBL isolates from the Shandong, Guangdong, Shaanxi, and Hainan provinces in China (Figure 3A). Among them, WM1 clustered closely with isolate HA3. Phylogenetic analyses based on the complete genome sequences of DNA-B of different SLCCNV isolates revealed WM1 grouped with GDBL, GDHY, BLDG, SDSG, SLCCNV-SDSGC, SLCCNV-SDZBZ, and SX01 isolates from the Guangdong, Shandong, and Shaanxi provinces in China (Figure 3B). Among them, WM1 clustered closely with GDBL, GDHY, and BLDG isolates.

### 2.4. The Infectious Clone of Isolate WM1 Can Systematically Infect Watermelon

**Figure 4:**
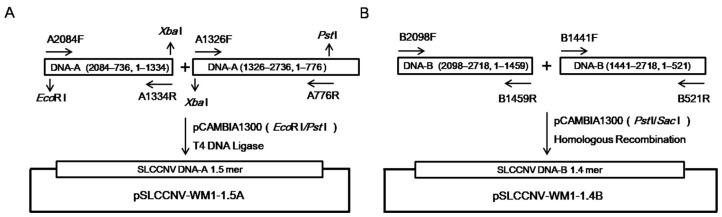
Strategies for construction of the infectious clone of SLCCNV WM1 isolate. DNA-A and DNA-B components of isolate WM1 were separated into two parts for amplification, and then a DNA-A infectious clone was constructed through enzyme digestion and ligation (**A**), while a DNA-B infectious clone was constructed through homologous recombination (**B**).

**Figure 5:**
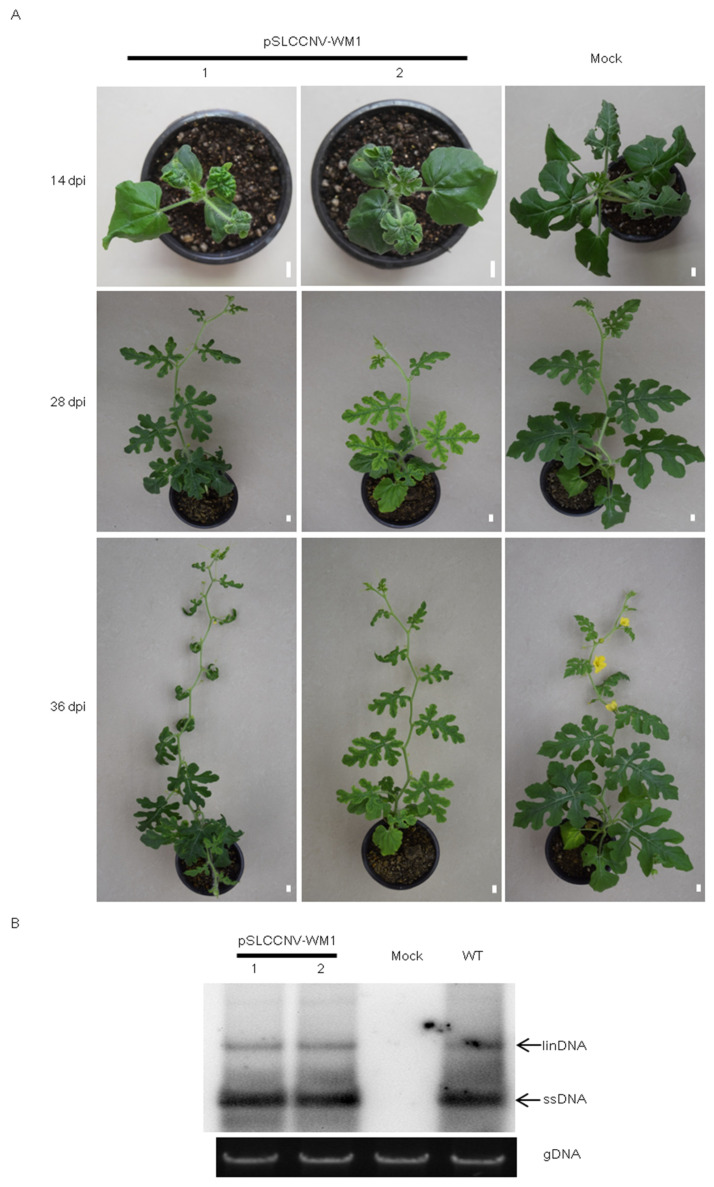
The infectivity of the infectious clone of SLCCNV WM1 isolate on watermelon. (**A**) Symptom development on watermelon inoculated with the cloned SLCCNV WM1 at 14 days post-inoculation (dpi), 28 dpi, and 36 dpi. (**B**) Southern blot analysis of the diseased leaves of watermelon inoculated with the cloned SLCCNV WM1 at 36 dpi. WT indicates wild type collected from the field. linDNA indicates linear DNA, ssDNA indicates single-stranded DNA, and gDNA indicates genomic DNA. Scale bars = 1 cm.

The infectious clone of isolate WM1 was constructed (Figure 4A,B) and inoculated to watermelon plants using an *Agrobacterium*-mediated method. Compared with the mock-inoculated plants, the growth of pSLCCNV-WM1-inoculated plants was slower, with small and curly leaves, accompanied by the emergence of dark green and light green mixed mosaic symptoms (Figure 5A). With increasing time since inoculation, the symptoms of the upper leaves of pSLCCNV-WM1-inoculated plants were more severe, and some plants gradually developed yellow–green mosaic symptoms (Figure 5A). The diseased leaves were collected and detected by Southern blot. The results confirmed that the presence of SLCCNV in pSLCCNV-WM1-inoculated plant leaves but not in mock-inoculated plant leaves (Figure 5B). Through PCR detection, the infection rate of pSLCCNV-WM1 was all 100% (12/12, 8/8, 8/8) in three independent assays.

### 2.5. The Infectious Clone of Isolate WM1 Can Also Systematically Infect Melon, Squash, Pumpkin, Wax Gourd, Cucumber, and N. benthamiana

**Figure 6:**
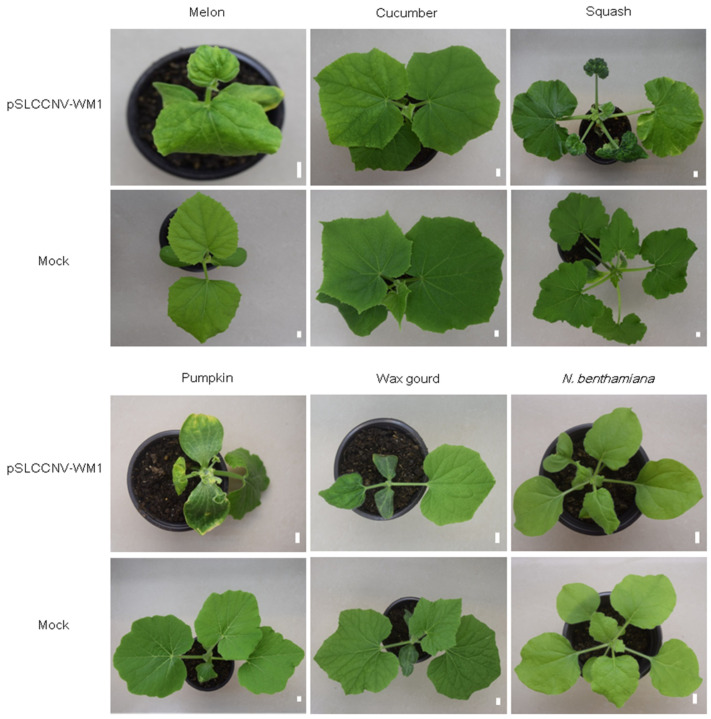
Symptom development on melon, cucumber, squash, pumpkin, wax gourd, and *N. benthamiana* inoculated with the infectious clone of SLCCNV WM1 at 10 dpi, 19 dpi, 14 dpi, 14 dpi, 14 dpi, and 14 dpi, respectively. Scale bars = 1 cm.

To assess the infectivity of the cloned SLCCNV WM1 in other crops, agroinoculation was performed on melon, cucumber, squash, pumpkin, wax gourd, and *N. benthamiana*. About 2 weeks after inoculation, the upper systemic leaves of melon, squash, pumpkin, wax gourd, and *N. benthamiana* inoculated plants developed curled and/or mosaic symptoms (Figure 6). At 19 dpi, 1 of 15 inoculated plants for cucumber cv. Jinyan 4 exhibited yellowing symptoms (Figure 6). The virus infection on tested plants was detected by PCR, and the infection rate of pSLCCNV-WM1 was 100.0% (18/18) for melon; 100.0% (18/18) for squash; 100.0% (8/8) for pumpkin; 100.0% (15/15) for wax gourd; 100.0% (9/9) for *N. benthamiana*; and 20.0% (3/15) for cucumber, respectively.

### 2.6. Progeny Virions Derived from the Cloned WM1 Isolate Can Be Transmitted by Whiteflies Rather than Mechanical

To assess whether progeny virions derived from the cloned WM1 isolate could be transmitted by whitefly, a whitefly transmission experiment was performed on healthy watermelon plants. Three weeks later, whitefly-inoculated watermelon plants developed curling and mosaic symptoms (Supplementary Figure S1A). PCR results showed that the transmission efficiency of two independent assays were 100% (8/8) and 80% (12/15), respectively, indicating that progeny virions derived from the cloned WM1 isolate was fully competent for whitefly transmission and symptom development.

To assess the mechanical transmission capability of progeny virions derived from the cloned WM1 isolate, the mechanical inoculation experiment was performed on both of healthy *N. benthamiana* and watermelon plants. However, by 21 dpi, all the inoculated plants, including 15 *N. benthamiana* plants and 36 watermelon plants, did not show any symptoms (Supplementary Figure S1B,C). PCR detection further confirmed that the inoculated plants did not contain the virus, and the infection rate was 0/15 for *N. benthamiana* and 0/36 for watermelon. This indicates that progeny virions derived from the cloned WM1 isolate cannot be transmitted through a mechanical mode of infection.

### 2.7. The Infectious Clone of Melon Isolate SLCCNV-HN Can Systemically Infect Melon and Watermelon, but Exhibits Lower Pathogenicity in Watermeon Compared to the WM1 Isolate

To assess the infectivity of the melon isolate SLCCNV-HN, we inoculated melon and watermelon with the infectious clone of SLCCNV-HN [20] and compared them with the plants inoculated with the cloned WM1 isolate. At 14 dpi, all melon plants inoculated with the cloned WM1 isolate or the cloned SLCCNV-HN developed symptoms, as did all watermelon plants inoculated with the cloned WM1 isolate (Table 2). However, only 12.5–50% of watermelon plants inoculated with the cloned SLCCNV-HN exhibited symptoms (Table 2). By 21 dpi, the disease incidence rate of SLCCNV-HN inoculated plants remained at 16.7–50% (Table 2). PCR detection revealed a 100% infection rate in both SLCCNV-HN-inoculated and WM1-inoculated plants (Table 2), indicating that a proportion of SLCCNV-HN-infected watermelon plants exhibited latent infection characteristics—being virally infected without manifesting conspicuous symptoms.

## 3. Discussion

In nature, SLCCNV can infect several crops in the Cucurbitaceae and Solanaceae families, such as pumpkin [18], squash [32], and wax gourd [33] in India, as well as melon [20], *Bolbostemma paniculatum* [23], tomato [21], and wild eggplant [22] in China. This study confirms that SLCCNV can infect watermelon, indicating that the host range of SLCCNV is continuing to expand. As is well known, SLCCNV is transmitted by whiteflies rather than sap, and the results of this study on the transmission ability of watermelon isolate are consistent with this. Whitefly is a destructive pest of vegetables worldwide, particularly as it serves as a vector of 212 plant viruses belonging to 5 genera, according to the data of the International Committee on Taxonomy of Viruses (ICTV), as of 2013 [34]. For cucurbits-infecting viruses, in addition to SLCCNV, the begomovirus ToLCNDV as well as the criniviruses cucurbit chlorotic yellows virus (CCYV) [34] and cucurbit yellow stunting disorder virus (CYSDV) [35] can also be transmitted by whiteflies. The widespread use of facility cultivation in the annual production of horticultural crops provides favorable living and breeding conditions for whiteflies and makes it possible for viral diseases to occur and spread. In this study, SLCCNV watermelon isolate was furthered confirmed to be able to infect other hosts such as pumpkin, squash, wax gourd, and melon. Therefore, the infection of SLCCNV in watermelons may be due to the virus being transmitted by whiteflies from other hosts. As described above, special attention should be paid to controlling the occurrence of whiteflies throughout the entire growth period of crops, preventing large-scale outbreaks of whiteflies, and setting up real-time monitoring of vector transmission and virus infection trends in crops in order to minimize the spread of SLCCNV.

SLCCNV has occurred in India [18], Pakistan [36], China [20], the Philippines [37], East Timor [38], Thailand [17], and Vietnam [39], and evolutionary analysis of SLCCNV isolates from different countries showed a clear geographical distribution. For example, isolates from the same country such as India, Vietnam, Thailand, etc., were more closely related than isolates from different countries [20,32], and the genetic relationship between isolates from neighboring countries such as India and Bangladesh, or China and Vietnam, is closer than that between distant countries such as India and Vietnam [22,40]. From the phylogenetic analyses of this study, isolates from China, Cambodia, Thailand, and Vietnam clustered together on one branch, with the closest phylogenetic relationship, while isolates from Indonesia and India clustered on other branches, with distant phylogenetic relationships. According to the species demarcation criteria of the genus *Begomovirus*, the ICTV *Geminiviridae* study group has proposed that a 91% and 94% pairwise sequence identity between viruses be used as thresholds for distinguishing different species and strains [30,41]. The identity analysis result of this study showed that the DNA-A sequence identity of isolate WM1 with other 14 isolates from China, Cs1 from Cambodia, KN44 from Thailand, and Hanoi from Vietnam were all higher than 94%, indicating that isolate WM1 belongs to the same strain as these isolates. However, the identity of isolate WM1 with WMK (93.5%) and SLCCV-[BASq-17] (93.3%) isolates from Indonesia, as well as KP1 (91.3%) and SLCCNV-ZUB1 (91.4%) isolates from India, were between 91 and 94%, indicating that isolate WM1 and these isolates belong to different strains of the same species. The above identity analysis results are consistent with the results of phylogenetic analysis in this study, suggesting the phylogenetic relationship of SLCCNV isolates were positively correlated with their geographical distance, which is also consistent with previous reported results [20,22,32,40].

From an evolutionary perspective, neither previous studies nor this study have found a clear correlation between the evolution of different isolates of SLCCNV and their hosts [20,32], and the inoculation results of this study did not reveal any specificity of SLCCNV watermelon isolates in infecting natural or experimental hosts. At present, there are multiple SLCCNV isolates from the same host in different countries, such as pumpkin isolates from India [18], East Timor [38], and Thailand [17], and wax gourd isolates from Thailand [42] and India [19]. Also, there are multiple SLCCNV isolates from different hosts in the same country, such as pumpkin [18] and wax gourd [19] isolates from India, and melon [20], tomato [21], and wild eggplant [22] isolates from China. Among them, the SLCCNV wax gourd isolates from Thailand and India have been confirmed to belong to divergent strains [19], consistent with the sequence analysis results of this study. Wu et al. [20] constructed an infectious clone of the melon isolate SLCCNV-HN. To clarify the pathogenicity of SLCCNV-HN in watermelon, we inoculated watermelon plants with a SLCCNV-HN infectious clone and observed that it could also infect watermelon. However, compared to the watermelon isolate clone obtained in this study, SLCCNV-HN exhibited lower pathogenicity in watermelon, with an incidence rate equal to or lower than 50%. These results indicate that SLCCNV-HN shows better adaptation to its natural host (melon) than to the experimental host (watermelon), suggesting that the watermelon isolate obtained in this study may possess specific host-adaptive evolutionary traits in watermelon. Future systematic studies on host-related molecular pathogenicity and molecular evolutionary mechanisms between these two isolates are warranted to clarify virus–host interactions and enhance our understanding of viral evolution.

In this study, we confirmed that SLCCNV isolate WM1 is responsible for yellow mosaic leaf curl disease in watermelon, using an infectious clone, and comprehensively characterized the DNA-A and DNA-B genomes of the isolate. SLCCNV was reported to infect watermelon in Indonesia in 2019, but the study did not conduct Koch’s postulates verification or perform whole-genome characterization analysis [24]. It is worth noting that the DNA-A complete genome sequence (OQ123829.1) of isolate WMK obtained from watermelon diseased leaves collected in Indonesia in 2021 could be retrieved from the NCBI database, but no corresponding DNA-B component sequence was found. This could be due to incomplete sequence information in the database or reflect the unique genetic characteristics of the WMK isolate itself. The sequence identity and phylogenetic analysis of this study indicated that the isolates WM1 and WMK belong to different strains of the same species (Table 1 and Figure 3A), and isolate WM1 is proposed to belong to the SLCCNV/CN strain according to the strain demarcation criteria of begomoviruses [30]. To the best of our knowledge, this is the first report of a natural infection of SLCCNV on watermelon in China, and the first complete report on the molecular characteristics of SLCCNV/CN watermelon isolates in the world. This study lays a foundation for subsequent research on the viral pathogenicity or host resistance and prepares strategies for the prevention and control of this virus disease. For instance, the infectious clone obtained in this study provides an important tool for future study on the function of viral genomes and the development of watermelon varieties resistant to SLCCNV infection. In the future, we will utilize this infectious clone to screen disease-resistant varieties to address the occurrence of this disease.

## 4. Materials and Methods

### 4.1. Materials

In October 2023, watermelon leaves displaying mosaic, yellowing, and curling in plastic greenhouses were sampled for testing in Ningbo, Zhejiang Province.

### 4.2. Next-Generation Sequencing (NGS) and Sequence Assembly

Genome DNA was extracted from the samples using MonPure™ Universal Genome DNA Kit (Monad, Suzhou, China) and was accurately quantified using a Qubit 3.0 Fluorometer. Subsequently, the DNA was fragmented to approximately 350 bp using a Covaris M220 sonicator (Covaris, Woburn, MA, USA). Then, DNA fragments were endpolished, A-tailed, and ligated with the full-length adapter for Illumina sequencing (Illumina, San Diego, CA, USA), followed by further PCR amplification. The quality of the constructed libraries was assessed using an Agilent 2100 Bioanalyzer (Agilent, Santa Clara, CA, USA) and quantitative PCR (Roche, Mannhein, Germany). Qualified libraries were sequenced on an Illumina NovaSeq 6000 system (Illumina) using a paired-end 150 bp (PE150) sequencing mode. The raw sequencing data were subjected to quality control using the fastp software (v0.21.0) with default parameters [43]. Subsequently, the watermelon genome (acc: GCA_029034555.1) KOR._cv.242 and the human reference genome (hg38) were employed as reference databases for the removal of host species sequences. The final filtered clean reads were assembled de novo using the SPAdes software v3.0.1 [44]. The resulting contigs were annotated by searching the NCBI NT database using Megablast (https://blast.ncbi.nlm.nih.gov/Blast.cgi, accessed on 19 January 2024) to identify segments of the target virus.

### 4.3. PCR Identification

Based on the results of NGS, two pairs of specific primers A304F/A1848R and B1092F/B2180R (Supplementary Table S1) for amplification of SLCCNV DNA-A and DNA-B were designed, and the samples were detected by PCR amplification using 2 × Rapid Taq Master Mix (Vazyme, Nanjing, China). PCR products were separated by electrophoresis on 1.2% agarose gel.

### 4.4. Amplification of Complete Genome Sequences of SLCCNV Isolates

Based on the results of NGS, another two pairs of specific primers A1326F/A776R and B2098F/B1459R (Supplementary Table S1) for amplification of SLCCNV DNA-A and DNA-B were designed. The primers A304F/A1848R and A1326F/A776R were used to amplify the complete sequences of DNA-A genome of SLCCNV isolates from three samples, and the primers B1092F/B2180R and B2098F/B1459R were used to amplify the DNA-B complete sequences by using 2 × Phanta Max Master Mix (Vazyme). PCR products were separated by gel electrophoresis and sequenced.

### 4.5. Sequence Analysis

Complete genome sequences of DNA-A and DNA-B components of three watermelon isolates (WM1, WM2, WM3) were assembled by CAP3 [45], and they were compared with that of other SLCCNV isolates downloaded from the GenBank database by using ClustalX 1.83 [46], and genome-wide nucleotide sequence consistency analysis was performed with the BioEdit version 7.0.9.0 [47]. Phylogenetic analyses were performed using MEGA X by the neighbor-joining method with 1000 bootstrap replicates [30].

### 4.6. Construction of Infectious Clone

To determine the infectivity of SLCCNV watermelon isolate, the infectious clone of isolate WM1 was constructed. The primers A2084F/A1334R and A1326F/A776R were used to amplify the 1987 bp and 2187 bp fragments of DNA-A, respectively, and then digested with *Eco*R I/*Xba* I and *Xba* I/*Pst* I, respectively. The two digested fragments were ligated with pCAMBIA1300 linearized with *Eco*R I/*Pst* I, to generate the infectious clone of DNA-A of isolate WM1 containing 1.5-mer DNA-A tandem repeat (1.5A), named pSLCCNV-WM1-1.5A. The primers B2098F/B1459R and B1441F/B521R were used to amplify the 2080 bp and 1799 bp fragments of DNA-B, respectively. The two fragments were ligated with *Pst* I/*Sac* I-digested pCAMBIA1300 using homologous recombination strategy, to generate the infectious clone of DNA-B of isolate WM1 containing 1.4-mer DNA-B tandem repeat (1.4B), named pSLCCNV-WM1-1.4B. pSLCCNV-WM1-1.5A and pSLCCNV-WM1-1.4B was confirmed by sequencing.

### 4.7. Agrobacterium-Mediated Inoculation

To assess the infectivity of the cloned SLCCNV WM1, pSLCCNV-WM1-1.5A, and pSLCCNV-WM1-1.4B were transformed into *Agrobacterium tumefaciens* GV3101, and agroinoculation was performed on watermelon (*Citrullus lanatus*) cv. Zhengkang 2, melon (*Cucumis melo.*) cv. Xinmiza 11, cucumber (*Cucumis sativus*) cv. Jinyan 4, squash (*Cucurbita pepo*) cv. Zhenyu 358, pumpkin (*Cucurbita moschata*) cv. Lvbeibei, wax gourd (*Benincasa hispida*) cv. Fenpidonggua, and *N. benthamiana* plants with bacterial suspensions mixed at a 1:1 ratio, which was detailed described in Liu et al. [48]. To assess the infectivity of the cloned SLCCNV-HN (Provided by Huijie Wu) [20], agroinoculation was performed on watermelon (*Citrullus lanatus*) cv. Zhengkang 2 and melon (*Cucumis melo.*) cv. Xinmiza 11.

### 4.8. Whitefly Transmission and Mechanical Inoculation

To assess the whitefly transmissibility of progeny virions from the cloned SLCCNV WM1, non-viruliferous whitefly was transferred to diseased watermelon plants inoculated with the cloned SLCCNV WM1 for a 7-day acquisition. Subsequently, the whiteflies were transferred to healthy watermelon plants for a 7-day inoculation. Then, the whiteflies were eliminated by spraying insecticides. After 3 weeks, upper uninoculated leaves were sampled and virus detection was performed by PCR.

To assess the mechanical transmissibility of progeny virions from the cloned SLCCNV WM1, the diseased watermelon leaves inoculated with the cloned SLCCNV WM1 at a ratio of 1:10 (mass-to-volume) were collected and homogenized in 1 × PBS buffer. This was inoculated on *N. benthamiana* and watermelon leaves by sprinkling with silicon dioxide powder as a carrier. After 3 weeks, upper uninoculated leaves were sampled and virus detection was performed by PCR.

### 4.9. Southern Blot Analysis

Partial sequence of *CP* gene of SLCCNV WM1 was amplified using primers A304F/A878R (Supplementary Table S1). The PCR product was labeled with digoxigenin (DIG) using DIG-High Prime (Roche) to generate the probe for Southern blot analysis. Genomic DNA was extracted from the systemic leaves of the inoculated plants, separated on 1.2% agarose gel, and then transferred to the Hybrid-N membrane (Amersham Biosciences, Little Chalfant, UK). Further analysis was performed to detect SLCCNV according to the instructions of the Detection Starter Kit I (Roche).

## 5. Conclusions

In this study, we identified that the pathogen causing mosaic, yellowing, and curling of the leaves of watermelon in Ningbo was a strain of SLCCNV/CN in the species *Begomovirus cucurbitachinaense* through PCR and NGS. Subsequently, we constructed an infectious clone of the isolate and its pathogenicity in watermelon, melon, squash, pumpkin, wax gourd, cucumber, and *N. benthamiana* was verified. The clone was fully infectious and showed identical properties to the watermelon isolate collected in the greenhouse. The clone will be an important tool for future studies on the functions of viral genomes and developing a watermelon variety resistant to SLCCNV infection.

## Figures and Tables

**Table 1:** The identity of nucleotide and amino acid sequences between SLCCNV WM1 and other isolates.

1. Isolate	2. Nucleotide Sequence Identity/%		3. Amino Acid Sequence Identity/%								
	DNA-A (GenBank Accession)	DNA-B (GenBank Accession)	AV1	AV2	AC1	AC2	AC3	AC4	AC5	BC1	BV1
BLDG	96.7 (MW389927.1)	97.9 (MW389928.1)	99.2	95.6	96.9	92.5	94.8	-	92.4	82.7	98.5
FSBG	97.9 (MW389923.1)	93.9 (MW389924.1)	97.6	97.8	98.0	94.0	94.1	-	95.9	88.5	91.7
GDBL	96.6 (MW389921.1)	98.1 (MW389922.1)	98.8	88.0	91.9	66.4	94.1	-	91.2	95.3	99.6
GDXW	98.3 (MW389919.1)	-	99.6	91.3	98.3	94.7	94.1	-	98.8	-	-
GDHY	98.2 (MW389917.1)	98.3 (MW389918.1)	99.2	97.8	98.0	94.0	94.1	-	98.2	79.3	98.8
Guangxi2017	94.8 (MG525551.1)	86.3 (MG525552.1)	98.4	-	94.7	88.8	92.6	-	87.7	94.3	90.6
HA3	99.2 (KF184993.1)	-	98.8	99.2	98.8	97.7	98.5	-	97.6	-	-
Hn	98.1 (MF062251.1)	93.1 (MF062252.1)	99.6	97.8	98.0	94.7	94.1	91.3	98.2	98.2	95.1
HN	98.1 (HM566112.1)	94.7 (HM566113.1)	99.6	99.2	97.2	92.5	93.3	-	99.4	98.9	94.7
SDSG	98.3 (MZ682117.1)	97.3 (OK236348.1)	99.6	97.8	98.0	94.7	94.1	96.5	98.8	99.2	96.6
SLCCNV-SDSGC	98.2 (OM258181.1)	97.4 (OM258182.1)	99.6	97.8	98.3	93.2	94.1	96.5	98.8	99.6	97.0
SLCCNV-SDZBZ	98.0 (OM258179.1)	97.6 (OM258180.1)	99.6	97.1	98.0	93.2	92.6	94.8	98.8	99.6	97.7
SX01	98.2 (OM100574.1)	97.3 (OM100575.1)	99.6	97.1	98.3	94.0	94.1	96.5	97.6	99.6	97.3
YN5947	94.4 (MK626660.1)	89.5 (MK626666.1)	80.4	-	72.6	89.5	92.6	89.6	71.4	95.3	89.1
J1	88.8 (MN594504.1)	81.5 (MN594505.1)	93.3	55.7	93.3	76.1	81.6	81.0	-	92.9	86.1
KP1	91.3 (KF188433.1)	81.6 (KJ004521.1)	95.3	66.6	94.4	83.5	88.9	82.7	-	90.4	83.2
SLCCNV-[PumIARI]	89.6 (JN587811.1)	84.3 (JN624306.1)	93.7	66.6	91.9	80.5	86.2	-	68.0	97.1	87.6
SLCCNV-ZUB1	91.4 (OR208615.1)	82.9 (OR208614.1)	96.0	66.6	93.6	81.3	88.9	79.3	70.3	97.1	88.0
Cs1	94.7 (MT682356.1)	89.8 (MT682357.1)	96.8	88.6	95.2	88.8	92.6	-	87.2	95.7	90.2
Hanoi	95.6 (KC857509.1)	91.9 (KC857510.1)	98.4	97.1	97.2	90.2	93.3	-	91.8	98.2	92.9
KN44	95.8 (MK978176.1)	89.5 (MK978177.1)	98.4	97.1	95.8	91.0	91.9	-	-	95.7	88.4
WMK	93.5 (OQ123829.1)	-	98.4	81.3	95.8	82.8	89.7	93.1	-	-	-
SLCCV-[BASq-17]	93.3 (LC511776.1)	84.6 (LC511781.1)	96.8	75.3	93.3	86.5	90.4	84.4	-	95.3	89.5

**Table 2:** Inoculation results of SLCCNV-HN and SLCCNV-WM1 infectious clones on melon and watermelon.

Isolate	Crop	Repeat 1			Repeat 2			Repeat 3		
		Incidence Rate ^a^		Infection Rate ^b^	Incidence Rate		Infection Rate	Incidence Rate		Infection Rate
		14 dpi	21 dpi	PCR	14 dpi	21 dpi	PCR	14 dpi	21 dpi	PCR
SLCCNV-HN	Melon	6/6 (100%)	6/6 (100%)	6/6 (100%)	6/6 (100%)	6/6 (100%)	6/6 (100%)	6/6 (100%)	6/6 (100%)	6/6 (100%)
	Watermelon	1/8 (12.5%)	3/8 (37.5%)	8/8 (100%)	3/6 (50%)	3/6 (50%)	6/6 (100%)	1/6 (16.7%)	1/6 (16.7%)	6/6 (100%)
WM1	Melon	6/6 (100%)	6/6 (100%)	6/6 (100%)	6/6 (100%)	6/6 (100%)	6/6 (100%)	6/6 (100%)	6/6 (100%)	6/6 (100%)
	Watermelon	8/8 (100%)	8/8 (100%)	8/8 (100%)	6/6 (100%)	6/6 (100%)	6/6 (100%)	6/6 (100%)	6/6 (100%)	6/6 (100%)

^a^ Incidence rate is indicated as the proportion of symptomatic plants relative to the total number of inoculated plants. ^b^ Infection rate was assessed via PCR on samples collected at 21 dpi and is indicated as the proportion of PCR-positive plants relative to the total number of inoculated plants.

## Data Availability

All data are available in the manuscript and the Supplementary Materials.

## Supplementary Materials

The following supporting information can be downloaded at: https://www.mdpi.com/article/10.3390/ijms26094289/s1.
