# Supplementary material for: Molecular Characterization and Pathogenicity of Watermelon Isolates of Begomovirus cucurbitachinaense
Source: Int J Mol Sci. 2025 May 1;26(9):4289. doi: 10.3390/ijms26094289 (PMC12072825; doi:10.3390/ijms26094289)
Supplement: Supplementary file 1 [file ijms-26-04289-s001.zip › ijms-3601483-supplementary.pdf]

Supplementary Table S1 Primers used in this study

Primer name	Primer sequence
A304F	TCATTTCAACTCCCGCGTCG
A776R	CCAAGCTTGCATGCCTGCAGTGCTCGGTTCATTGT
A878R	CCTAACTAATGCTTGCTCCC
A1326F	GGTACCCGGGGATCCTCTAGAAAAGGTCTGGTGACG
A1334R	TGCCTGCAGGTCGACTCTAGAGGAACACGACATAATC
A1848R	CTAAGCCCGAGAGTATACAAT
A2084F	CAGCTATGACCATGATTACGAATTCATATTATGAAATTGCAAAACG
B521R	GCCAAGCTTGCATGCCTGCAGGCTGAATATCGTCTTCCCGG
B1092F	ATTCTCAATTCGTAACTGCATC
B1441F	GTGATGCCGATGGACCAGG
B1459R	CCTGGTCCATCGGCATCAC
B2098F	CATGATTACGAATTCGAGCTCCGTCGTTTGTGAGAGCGTAC
B2180R	GTCAATAAGAAATGATAACATGGG

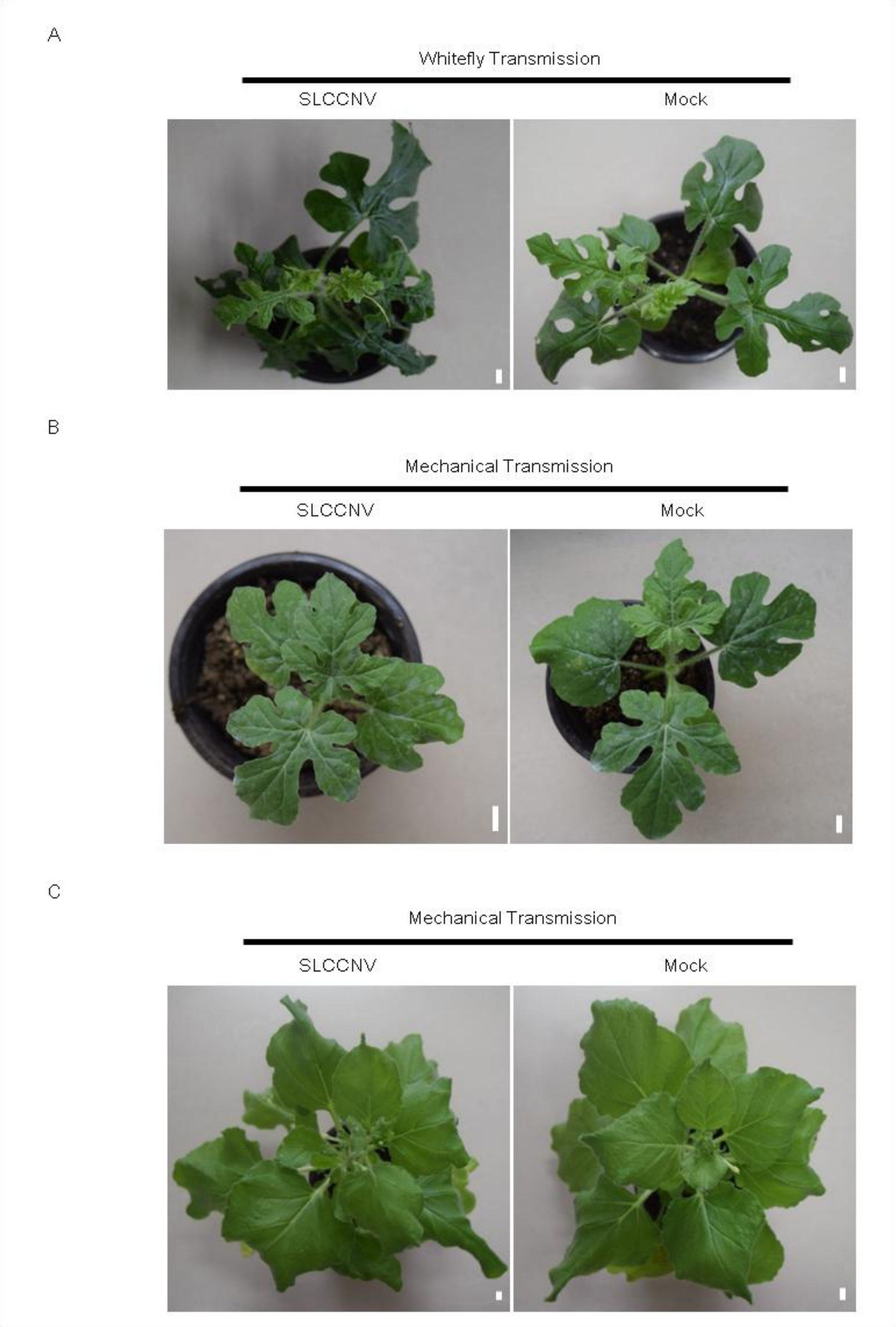

Supplementary Figure S1. Symptoms induced by whitefly transmission and mechanical transmission of progeny virions derived from the cloned SLCCNV WM1. A: Symptoms induced by whitefly transmission on watermelon at 21 dpi; B and C: Symptoms induced by mechanical transmission on watermelon and *N. benthamiana* at 21 dpi. Scale bars=1 cm.
